# By-Ways of Medical Relief

**Published:** 1911-11-18

**Authors:** 


### BYWAYS OF MEDICAL RELIEF.

(Contributions are invited to this column.)

### III.—Little Homes for Chronic Patients.

The little homes may either attempt to perform the same kind of work as that undertaken by ordinary hospitals, or they may assist the hospitals by receiving convalescent patients in early stages of recovery and thus make room for other sufferers, or lastly, and this is their largest sphere, they may aim at supplementing the work of hospitals by relieving persons ineligible for ordinary treatment. Among the cases most in need of help, yet impossible for hospitals to deal with, are the chronic and so-called incurables. Excluding consumptives, who now by common consent fall into a category of their own, there is a large class of helpless and infirm persons, disabled from work and in need of more attention than can usually be afforded them in a working-class home, even when such a home with relatives is open to them. The workhouse sick wards are the ordinary refuge for destitute chronic cases and it is very satisfactory to testify to the growing improvement in this aspect of Poor Law work. But manifestly the workhouse wards are not a fit resort for all such sufferers. The disabled nurse or governess, the clerk and skilled mechanic, hurled from the active currents of life by accident or disease, ought not to be compelled to associate with the ignorant or the vicious in the hour of adversity. The accommodation for incurable sufferers as well as for those who are doomed by their maladies to a year or two of inaction is quite inadequate. The process of procuring admission to the large institutions which meet their needs might daunt the strongest, and few in proportion can hope for any share in the benefits they offer. An extension of disablement aid is urgently called for, and it is quite possible that were such aid available many who now drift into the incurable stage might taste the joys of active work once more.

It is not an easy matter, however, to provide suitable accommodation even for a few chronic patients. Skilled nursing is an absolute necessity even when the chronic patients received are not in the acute stages of disease. Amateur attendance, however conscientious, falls short in a hundred directions of the efficiency which should be rigidly ensured to helpless patients taken out of their own homes. A higher standard altogether than that of the ordinary home should be aimed at by all who undertake to manage establishments for this class of sufferers. Privations which are borne very well among friends and relatives are a source of intense misery when encountered in a region of rules and restrictions. The happy-go-lucky atmosphere of the cottage or tenement room has in it something not uncongenial, and there are many uncovenanted mercies which contribute to ease the burden of pain. All then who take upon themselves the responsibility of alleviating the suffering of helpless patients must be prepared to accept the full onus of supplying the physical, mental, and moral needs of their patients. It is a campaign in which they embark, no mere tedious routine of mechanical services performed for somewhat self-centred individuals.

It must then be considered an axiom that however mild the nature of the chronic cases received and however few they may be in number, a trained nurse at the head of the home is essential. The construction of the house in which the patients are to be received is very important. Bedrooms on the ground floor for the paralysed and crippled, easy access to good lavatories, an airy sitting-room, facilities for an outdoor life whenever the climate permits—these are some of the considerations which should guide the manager in making choice of a suitable house. Unless these points can be satisfactorily dealt with, unless accommodation can be obtained which shall spare the attendants trouble and make the routine work of the establishment easy, it is better not to embark in the care of chronic patients.

From many months or years of suffering such patients often drift into a condition of auto-suggestion in respect of pain and helplessness, which it is easy to judge harshly as akin to shamming, though it may be all unconscious. The processes of the mind in chronic sufferers are very complicated, and it is no easy task to restore the mental equilibrium which has been sapped by a settled conviction of incurability. To deal successfully with patients of this description great tenderness must get to work with inspiring energy, and experience has abundantly proved that wonderful alleviations, if not cures, may be anticipated with wise treatment under good medical advice. Those who contemplate initiating work of this description may be advised that patients under forty show the best results, and it is better in any case not to allow the home to become altogether a resort for the aged, who require in many respects very different treatment. There is an unhappy tendency for physical ailments to assume baffling mental phases, and the element of causeless hostility to attendants or to other patients is to be guarded against. When it has once taken root it is a symptom that some change of residence is advisable, and even removal to workhouse wards may be a better alternative to permitting the miserable rancour which sometimes poisons such patients' minds and spoils the atmosphere of the home.

Private homes for the incurable exist, but are not easily discoverable. There are some under the charge of nuns and Anglican sisters. The Home of the Holy Rood at Worthing under the Wantage Sisters is one of the most beautifully ordered in all particulars which we have had the happiness to inspect, but its rapid development of late years is removing it out of the sphere of little homes. St. Cyprian's Home at Hammersmith is doing excellent work, and so is the Helena Home at Reading where patients have every alleviation which skill and good nursing can supply.

